# Accelerating the knowledge of Peruvian Chalcididae (Insecta, Hymenoptera, Chalcidoidea) with integrative taxonomy

**DOI:** 10.3897/BDJ.7.e35907

**Published:** 2019-12-04

**Authors:** Bruno Cancian de Araujo, Marcelo Teixeira Tavares, Thales Renan Aguiar Brotto, Juliana Martins Silva-Freitas, Max Estefani Vagner Santos, Pâmella Machado Saguiah, Stefan Schmidt

**Affiliations:** 1 SNSB-Zoologische Staatssammlung München, Munich, Germany SNSB-Zoologische Staatssammlung München Munich Germany; 2 Laboratório de Biodiversidade de Insetos, Universidade Federal do Espírito Santo, Vitória, Brazil Laboratório de Biodiversidade de Insetos, Universidade Federal do Espírito Santo Vitória Brazil

**Keywords:** Biodiversity, parasitoids, DNA barcoding, Peru, survey, species discovery

## Abstract

We present the first regional inventory of the fauna of Chalcididae in the Peruvian Amazon, with a nearly 6-fold increase in the number of species recorded for the country. A total of 418 specimens of Chalcididae were collected between 2000 and 2017 at the Panguana Reserve, Peruvian Amazon, 400 of which were obtained using Malaise traps and the remaining 18 specimens by canopy fogging. The morphological analyses indicated that these specimens represent 183 species of Chalcididae in 10 different genera, with 173 new to Peru and 134 potentially new species. We submitted 268 specimens, representing 167 species, to DNA barcoding. Of these, 141 specimens yielded sequences, 136 of them with a minimum of 300 bp. Sixty specimens were assigned a BIN by the Barcode of Life Database System (BOLD), resulting in 50 BINs. A cluster analysis of 138 individuals that yielded DNA sequences longer than 100 bp revealed 118 MOTUs (molecular operative taxonomic units), all of them highly congruent with the morphological data. Prior to the present study, 37 species in 9 genera of Chalcididae were known from Peru. With our results, this number was increased to 210 species in 13 genera. The present study is the result of a joint effort between the SNSB - Zoologische Staatssammlung München, Germany (ZSM) and the Insect Biodiversity Laboratory of the Universidade Federal do Espírito Santo, Vitória, Brazil (LaBI-UFES), intending to apply an accelerated taxonomic treatment of the Chalcididae of the Panguana reserve using traditional morphological approaches in combination with DNA barcoding. The complete molecular dataset and associated voucher information is publicly available through BOLD. The new species that were discovered as part of the study are being formally described elsewhere as part of taxonomic treatments of Neotropical and world generic revisions at LaBI-UFES.

## Introduction

Chalcididae is a medium-sized family with about 1,500 documented species in 87 genera and five subfamilies (Noyes 2018). Most individuals are between 1.5 and 24 mm in size (Tavares and Aquino 2014) and are recognised, amongst other characters, by the parallel inner margins of the compound eyes, rounded tegula, the lateral margin of the mesoscutum ∩-shaped and by their usually robust body with enlarged hind femora that are equipped with one or more teeth (or a comb of denticle) on the ventral margin.

Most of Chalcididae species are primary parasitoids of Lepidoptera and Diptera and a few species attack Coleoptera, Hymenoptera, Neuroptera or Strepsiptera (Tavares and Aquino 2014). Some species are secondary parasitoids (obligatory or facultative) through Hymenoptera (Dryinidae or Ichneumonoidea) and Diptera (Sarcophagidae or Tachinidae). The secondary hosts are Lepidoptera (many families), Coleoptera (Coccinellidae), Hemiptera (Auchenorrhyncha), Orthoptera (Acrididae) and Araneae (Theridiidae) (Tavares and Aquino 2014, Noyes 2018, Tavares et al. 2019). Although it is a family of parasitoid wasps that are moderately species-rich, the diversity of attacked hosts and of others biological aspects make the family contribute in a very diverse way to the structure and function of ecosystems. In addition, many species have shown effective or potential importance as natural enemies of pests (Delvare 2006).

Peru is one of the 17 megadiverse countries of the world (Mittermeier et al. 1999). However, only 38 species of Chalcididae have been recorded from the country so far (Noyes 2018), reflecting the lack of dedicated studies of chalcidoid fauna in that country (Table 1).

With the rapid decline and loss of biodiversity, mainly caused by deforestation and land fragmentation (Chavez 2013, Asner et al. 2017), studying and describing the biodiversity of these areas at an accelerated pace has become a challenge for taxonomists (Myers et al. 2000, Erwin et al. 2007). Integrative taxonomy can be a useful tool for the discovery and documentation of biodiversity, by combining morphological characters and DNA barcoding approaches, providing accessible and applicable data to support conservation.

The present study is the result of a joint effort of the SNSB-Zoologische Staatssammlung München, Germany (ZSM) and the Insect Biodiversity Laboratory at Universidade Federal do Espírito Santo, Vitória, Brazil (LaBI-UFES) that aimed at providing an accelerated taxonomic study of the Chalcididae of the Panguana research station (Huanuco province) using traditional morphological approaches in combination with DNA barcoding.

## Material and methods

The specimens were collected between 2000 and 2017 using Malaise traps (Townes 1972) placed at irregular intervals (representing around 500 trap-days) at the Panguana reserve, Peru (9.6137°S, 74.9356°W). Panguana is located at approximately 230 m elevation on the Yuyapichis river, occupying an area of 950 hectares (9.5 square kilometres) covered mainly by tropical primary forest, with an annual average temperature of 24.5°C and 2500 mm of precipitation distributed in about 180 days per year. Samples from sixteen Malaise traps from ten different years were sorted and Chalcididae extracted. Additional samples were obtained in December 2017 from canopy fogging (Floren 2010), resulting in 48 samples, of which eight samples contained specimens of Chalcididae that were included in our study.

### Workflow

After the signature of a Memorandum of Understanding (MoU) between UFES and ZSM, a joint effort was settled by BCA, SS and MTT to accelerate the work process by using the ZSM expertise in molecular biology and LaBI-UFES expertise in Chalcididae taxonomy. BCA was responsible for the general coordination, samples sorting and genera identification (one week for sorting and identification), SS was in charge of the molecular coordination and MTT was in charge of the taxonomic coordination. MTT selected the specialists for each genus found during the sorting process. The specimens were illustrated following the specialist's request and the images sent via the ZSM server. Over one thousand images were prepared and sent in two weeks. Since *Conura *represented almost 40% of all specimens, TRAB was sent to ZSM to accelerate the identification process (one month). In order to avoid shipping a large amount of material, only a few specimens, not identified by the images, were sent from Germany to Brazil according to the specialist's request. Each specialist made the first identification of the material and selected the specimens with priority on the molecular pipeline. The material was plated in ZSM, sent to CCDB and the results analysed by SS, who communicated with the specialists and coordinated new rounds of molecular analysis. This process took around two months and was important to avoid sequencing the same species several times, thereby saving money and time. BCA, MTT and SS coordinated, together with the specialists, the interpretation of the morphological and molecular results and the definition of the species lists. The specialists prepared the results and discussion for each genus under MTT coordination (one month). BCA, MTT and PMS prepared the backbone of the manuscript while SS wrote the methodology. BCA, MTT and SS wrote the general discussion. In the end, all authors made changes and suggestions in the entire manuscript. All this coordination was performed by using e-mail and message apps, allowing real-time discussion. The entire process, from the beginning of the samples sorting until the paper submission, including delays, took ten months.

### Taxonomic data assessment

The taxonomic data compilation for the named specimens was based mainly on Noyes (2018). The data for each species is listed ****in Table 1.

### Morphological analysis

Specimens were sorted to genus level at ZSM and then forwarded to taxonomic specialists at LaBI-UFES. Specimens for DNA barcoding were selected by the specialists based on morphological examination, aiming to obtain a set of specimens that reflected the species diversity. Long series of the same species were excluded to reduce cost and effort for the molecular analyses. Whenever possible, the specimens were identified to species level. Putative new species were flagged at this step. All specimens were imaged using a Nikon V1 camera attached to a Leica Z16 APO objective. Most specimens became part of revisional studies conducted as masters or doctoral theses at LaBI-UFES. The new species that were discovered will be formally described as part of the resulting taxonomic treatments of Neotropical and world generic revisions.

### DNA sequencing

Whole specimens were submitted to the Canadian Centre for DNA Barcoding (CCDB) in Guelph, Canada, for DNA extraction and sequencing. The voucher specimens were submitted to non-destructive DNA extraction and sequencing for subsequent preparation and morphological study. DNA extraction, PCR amplification and sequencing were conducted at the CCDB using standardised protocols (Ivanova et al. 2006, deWaard et al. 2008, Ratnasingham and Hebert 2007 http://ccdb.ca/). The 658 bp target region, starting from the 5’ end of the mitochondrial cytochrome c oxidase subunit I (COI) gene, includes the DNA barcode region for the animal kingdom (Hebert et al. 2003). The DNA extracts are stored at the CCDB. All specimen data are available through the Barcode of Life Database (BOLD) as a single, citable dataset (dx.doi.org/10.5883/DS-PECHAL1). The dataset includes collecting locality, geographic coordinates, elevation, collector, one or more digital images, identifier and voucher depository. Sequence data can be obtained through BOLD and include a detailed LIMS report, including chromatograms, primer information and access to trace files. The sequences are also available on GenBank (Accession nos. MK202011-MK202148). 

Data on genetic material contained in this paper and the Barcode of Life Database (BOLD) are published for non-commercial use only, according to the agreements with the country providing the analysed samples. Use by third parties for purposes other than non-commercial scientific research may infringe the conditions under which the genetic resources were originally accessed and should not be undertaken without obtaining consent from the corresponding author of the paper and/or obtaining permission from the original providers of the genetic material.

### Data analysis

Sequences were aligned using the BOLD Aligner (amino acid-based hidden Markov models). The analyses are based on sequences with a minimum length of 300 bp and < 1% ambiguous bases. Genetic distances and summary statistics were calculated using analytical tools in BOLD and are given as mean and maximum pairwise distances for intraspecific variation and as minimum pairwise distances for interspecific variations.

Sequence divergence statistics were calculated using the Kimura two-parameter model of sequence evolution (Kimura 1980). The Barcode Index Numbers (BINs) that are assigned by the BOLD system represent globally unique identifiers for clusters of sequences that correspond closely to putative species (Ratnasingham and Hebert 2013). For BIN assignment, a minimum sequence length of 500 bp is required and sequences between 300 and 500 bp can join an existing BIN, but will not create or split BINs. BINs provide an interim taxonomic system and a way to determine Molecular Operational Taxonomic Units (MOTUs), prior to detailed taxonomic studies including morphology.

Sequences between 100 and 499 bp that did not meet the requirements for BIN assignment were submitted to a cluster analysis that included all sequences with a length of at least 100 bp. The analysis tool is provided by the BOLD system and generates OTUs independent of the BIN registry, using the REfined Single Linkage algorithm (RESL – Ratnasingham and Hebert 2013). In the tables presenting Sample IDs and BINs, "N/A" was used for missing data.

## Results

**Figure 1.**
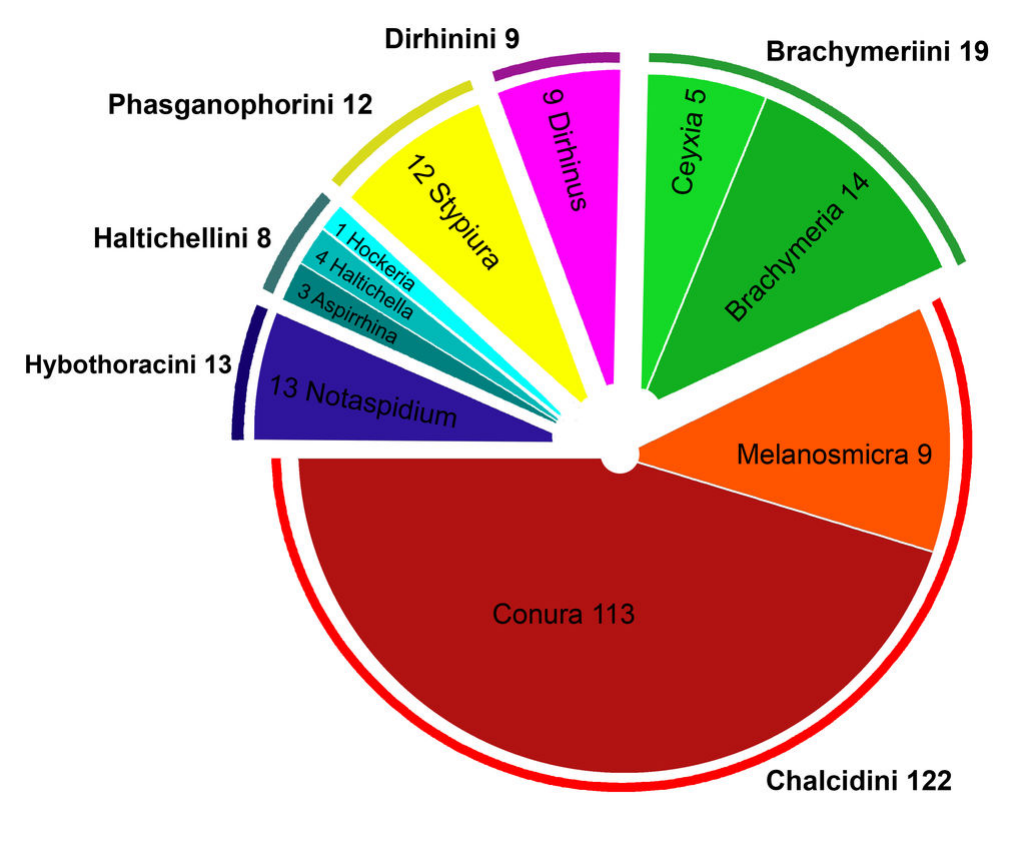
Proportion of Chalcididae species recovered in samples from Panguana station, Huánuco, Peruvian Amazon, per genera and tribes.

A total of 418 specimens of Chalcididae were morphologically examined. The malaise traps provided 400 specimens and canopy fogging, the remaining 18. The morphological analyses revealed 183 species of Chalcididae in ten genera (Fig. 1): 49 described species and at least 134 new species (Suppl. material 1). From a total of 76 described species found in the samples, only 10 species previously known to occur in Peru were recovered (Table 1), resulting in 66 new occurrence records for the country. Four genera (*Aspirrhina*, *Haltichella*, *Hockeria* and *Melanosmicra*) are also new distribution records for Peru, bringing the current total of Peruvian Chalcididae to 13 genera and 210 species (76 described and 134 new to science) (Suppl. material 1).

We submitted 268 of these 418 specimens representing 167 species to molecular analysis. Of those, 141 specimens yielded COI barcode region sequences, 136 of them with at least 300 bp. Sixty specimens were assigned a BIN by the BOLD system, resulting in 50 BINs. A cluster analysis with the 138 individuals that produced sequences longer than 100 bp revealed 118 putative OTUs (putative species) with almost full congruence with the morphological analyses performed (Table 2).

### *Conura *Spinola, 1837

**Figure 2.**
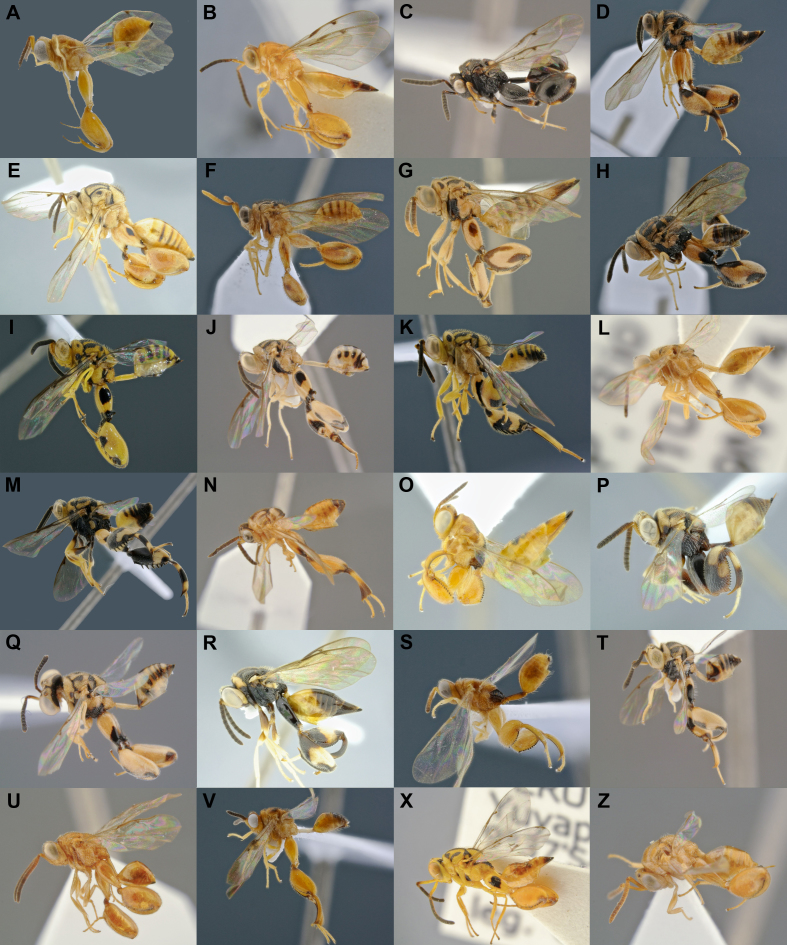
Diversity of *Conura *subgenera and species groups (Delvare 1992) and respective BINs. All specimens are females, except F and K: **A.** *Co. *(*Ceratosmicra*) *camescens*(*immaculata* group; BOLD:ADI0819); **B.** *Co.* (*Ce.*) sp77 (*onorei* group); **C.** *Co. *(*Ce.*) *dorsimaculata*(*side *group); **D.** *Co. *(*Conura*) *dares*(*dares*group); **E.** *Co. *(*Co.*) *nigrifrons*(*maculata *group; BOLD:ADE9829); **F.** *Co. *(*Co.*) sp90 (*vau*group); **G.** *Co. *(*Spilochalcis*) sp76 (*picta *group); **H.** *Co. *(*Sp.*) sp04 (*chrysomera *group; BOLD:ADI0628); **I.** *Co. *(*Sp.*) sp23 (*surumuae *group); **J.** *Co. *(*Sp.*) *vesicula*(*vesicula*group); **K**. *Co. *(*Sp.*) sp29 (*annulipes *group); **L**. *Co. *(*Sp.*) sp45 (*femorata*group); **M.** *Co. *(*Sp.*) *amoena*(*flava *group; BOLD:ADI0816); **N.** *Co. *(*Sp.*) sp63 (*pygmaea*group); **O.** *Co. *(*Sp.*) sp16 (*aequalis *group; BOLD:ADE9956); **P.** *Co. *(*Sp.*) sp17 (*arcuaspina *group; BOLD:ADE9958); **Q.** *Co. *(*Sp.*) sp68 (*contributa *group); **R.** *Co. *(*Sp.*) *santaremensis*(*discolor *group); **S.** *Co. *(*Sp.*) *adela*(*maculipennis*group; BOLD:ADI1049); **T.** *Co. *(*Sp.*) sp62 (*rasplusi*group); **U.** *Co. *(*Sp.*) sp44 (*rufodorsalis *group); **V.** *Co. *(*Sp.*) sp89 (*xanthostigma *group; BOLD:ADH8745) ; **X.** *Co. *(*Sp.*) *attacta*(*femorata*group); **Z.** *Co. *(*Ce.*) *immaculata*(*immaculata*group).

*Conura *was the most abundant and richest genus sampled with a total of 166 specimens sorted into 113 species, a trend found in other checklist papers of Chalcididae (Arias and Delvare 2003, Tavares and de Araujo 2007). It was the second most common and diverse genus in the fogging samples, with six specimens representing six species, (one formally described and five new species). *Conura* currently includes 301 valid species (Noyes 2018), with 8 recorded in Peru so far (Table 1). This study recovered two of those species, *Co. attacta* and *Co. immaculata* and added another 22, totalling 30 described species for the country (Fig. 2). At least 37 species obtained in this study are new species, which are being described in papers in preparation at LaBI-UFES. One species (*Co. *sp90) was represented by 11 specimens and another two (*Co. *sp3 and *Co.* sp35) by six specimens; however, 77% of the sampled species were singletons. Despite being the second most dominant genus in the fogging samples, only six species singletons were obtained.

*Conura* systematics is rather complicated, with three subgenera, three species complexes and 63 species groups (Delvare 1992). All subgenera and species complexes were represented in the samples, as well as 24 of the 63 species groups (Suppl. material 1). A total of 135 specimens, representing the majority of sampled species of *Conura, *were chosen for the molecular pipeline. Of those, 80 specimens produced sequences of at least 200 bp and were included in the cluster analysis (Table 3). The clusters presented an average of 13.51% nearest neighbour (NN) distance for the genus *Conura*. Comparing the subgenera separately, the average nearest neighbour (NN) distance is 8.32% for the subgenus Ceratosmicra, 24.63% for subgenus Conura and 12.48% for subgenus Spilochalcis. Thirty-seven BINs were associated with the specimens sequenced, all new to BOLD (Table 3).

**Figure 3.**
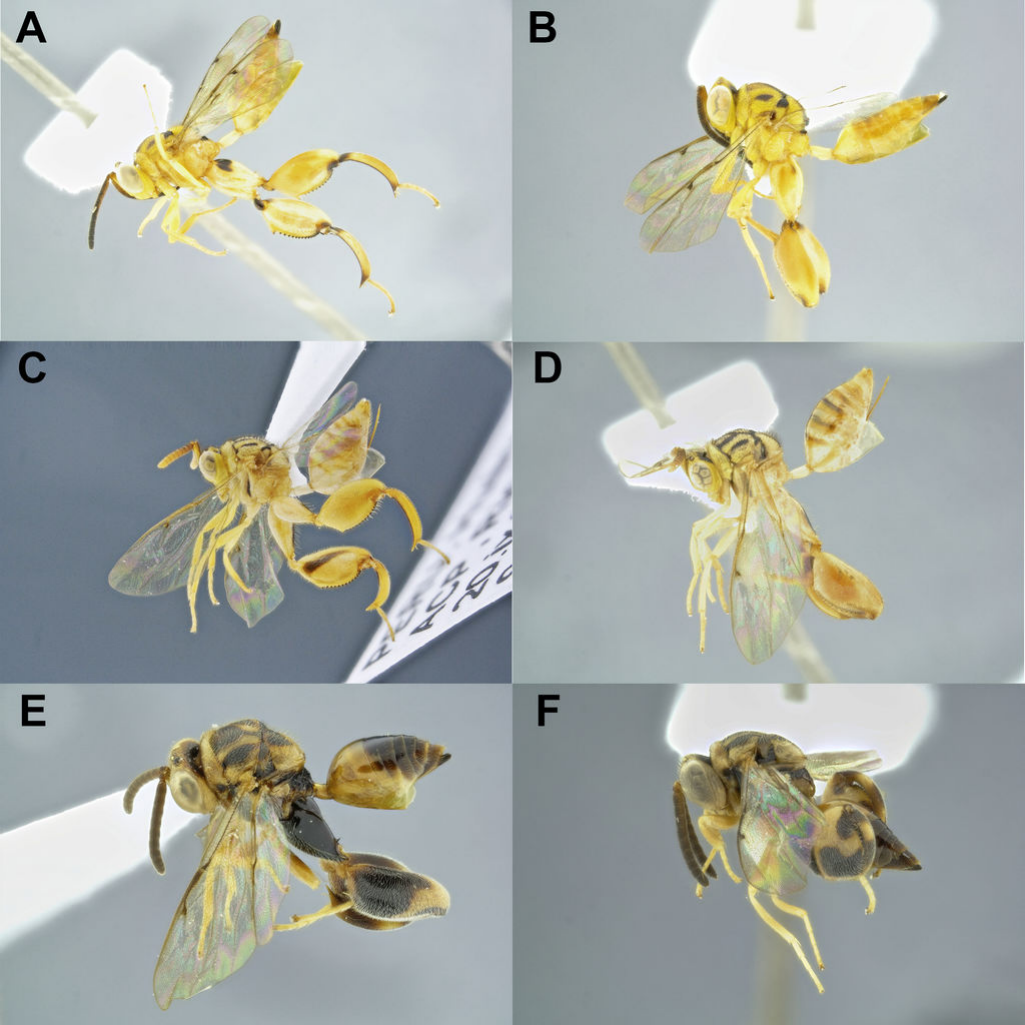
Species of *Conura *with morphologically indistinct specimens and two BINs, females: **A** and **B.** *Conura *(*Spilochalcis*) sp3**(*femorata*group*; ***A.** BOLD:ADE9827; **B.** BOLD:ADE9681); **C & D.** *Co.* (*Conura*) sp6 (*maculata *group; **C.** BOLD:ADI0818; **D.** BOLD:ADE9680); **E & F.** *Co. *(*Sp.*) sp18 (*discolor *group; **E.** BOLD:ADE9682; **F.** BOLD:ADE9822)*.*

Some species had specimens recovered in different BINs, but were morphologically indistinct at first analysis, such as *Co.* sp3, *Co. *sp6, *Co. *sp9, *Co. *sp12 and *Co. *sp18 (Fig. 3). Further morphological analyses did reveal differences within *Co. *sp9 and *Co. *sp12, but no significant differences were found amongst *Co.* sp3, *Co. *sp6 and *Co. *sp18. *Co. *sp9 and *Co. *sp12 were then divided into *Co. *sp9 and *Co. *sp89 and *Co. *sp12 and *Co. *sp90, respectively. *Co.* sp9 and *Co.* sp89 differ in number and size of metafemur teeth, the first one with 13 and the second with 18 smaller, very close together and the gaster of *C.* sp9 is higher than in *C.* sp89. *Conura* sp12 and *C.* sp90 were more similar and harder to split, the only clear difference between these species was the lower face strigate in *C.* sp12 and shallow fovea in *C.* sp90. This suggests that the DNA barcode may be a tool to refine taxonomy and even for detecting potential cryptic species of Chalcididae. The other three species remained morphologically indistinct, in spite of their different BINs. Perhaps further studies, including aspects of internal morphology, genitalia, biology and additional developmental data could help elucidating those cases.

**Figure 4.**
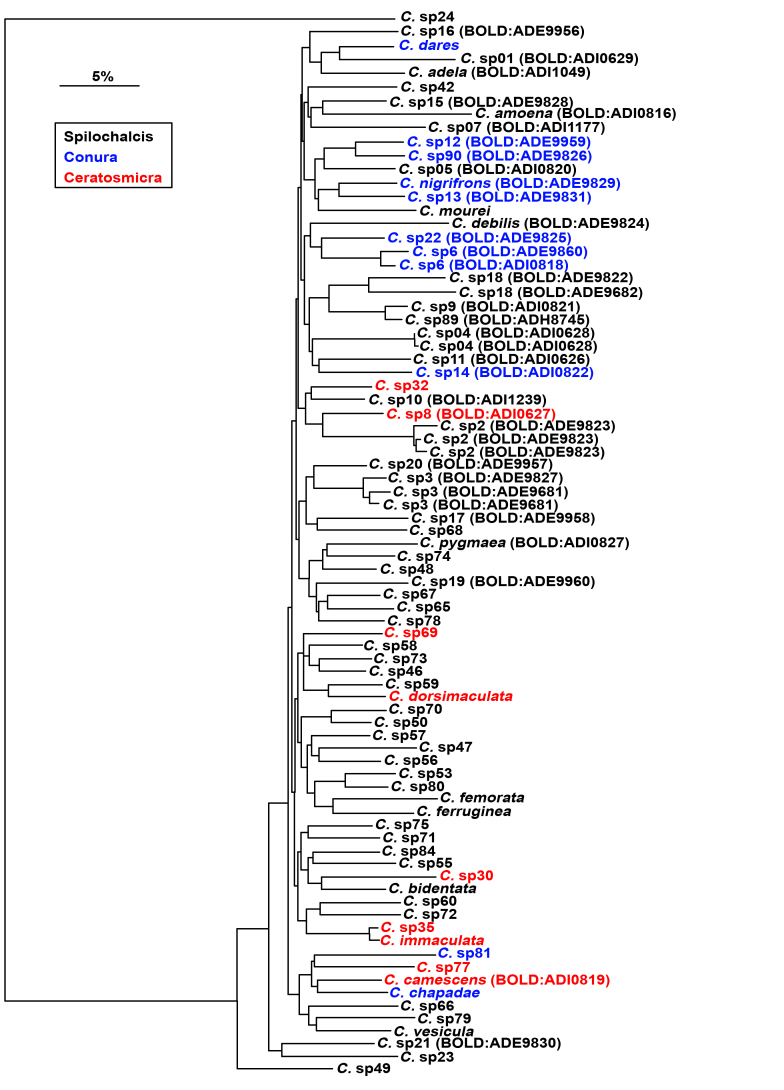
A neighbour-joining distance tree of the *Conura *specimens with sequences longer than 200 bp. The colours represent subgenera. Available BINs are presented between brackets before the OTUs.

Figure 4 shows a neighbour-joining distance tree (Fig. 4) with sequences longer than 200 bp and the subgenera of *Conura* highlighted with different colours. All subgenera, species complexes and several species groups were recovered as distinct groups. However, these results may suggest that the relationships within *Conura* do not fully correspond to the hypotheses previously proposed by Delvare 1992. Some morphology-based phylogenetic studies, developed at LaBI-UFES, also refute Delvare 1992. However, it remains to be demonstrated to what extent the fast-evolving COI gene is suitable for assessing higher level phylogenetic relationships within Chalcididae.

### *Melanosmicra *Ashmead, 1904

**Figure 5.**
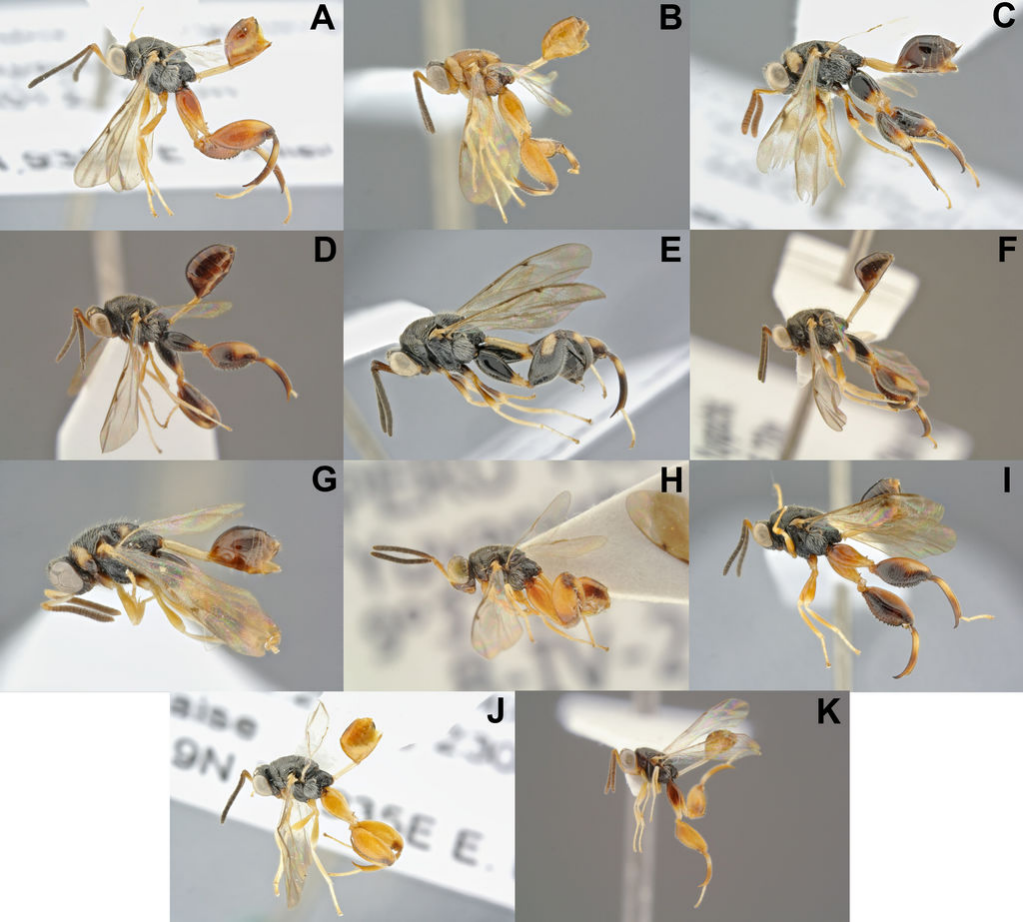
Diversity of *Melanosmicra*. **A.** *M. areta*, female (BOLD:ADF1337); **B.** *M. carenata*, female; **C.** *M. flavicollis*, female**(BOLD:ADE9942); **D.** *M. gracilis*, male; **E.** *M. immaculata*, male (BOLD:ADF0815); **F.** *M. nigra*, male; **G.** *M. rugosa*, female; **H.** *M. tricolor*, male; **I.** *M. *sp1, female (BOLD:ADF1769); **J.** *M. *sp2, female; **K**. *M. *sp3, male.

*Melanosmicra* is the only genus of the subfamily Chalcidinae found as new to Peru in this work. The number of specimens obtained with Malaise trap sampling (64 specimens or 15% of the total) was expressive (Table 4). Those specimens belong to 11 species, with three new species (Fig. 5). From this total, 21 specimens, representing all species found, were submitted to the molecular pipeline, producing 15 sequences longer than 100 bp, which corresponded to four different BINs. The molecular findings were congruent with the morphological results. The numbers here reported put Peru as the second country in terms of number of species of *Melanosmicra,* with eight described species and three species new species, after Brazil, with 13 species recorded (Navarro-Tavares and Tavares 2008).

### *Brachymeria *Westwood, 1829

**Figure 6.**
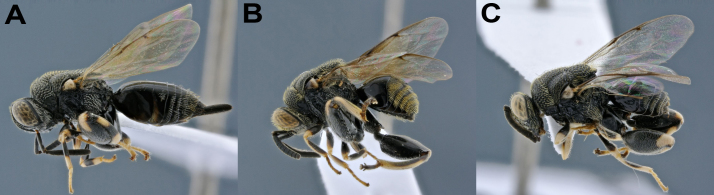
Diversity of the *Brachymeria *described species: **A.** *B. caudigera*, female; **B.** *B. mnestor*, male; **C.** *B. pandora*, male.

*Brachymeria* was represented by 67 specimens belonging to 14 species, 11 new species and three described (Table 5). Only the singleton *B. *sp 1 was collected in the fogging samples; the remainder were captured by Malaise trap. *Brachymeria* *mnestor* and *B*. *pandora* are frequently collected in the Neotropics. *B. caudigera* is very uncommon and it had been recorded so far only from the type locality (Jataí, State of Goiás, Brazil) (Fig. 6). Only four *Brachymeria* species had been previously recorded from Peru (Table 1). Therefore, with the records in this study, this number is increased to 18.

Seventeen specimens, representing eleven species, were selected to be submitted to molecular pipeline and produced five sequences larger than 500 bp, resulting in four different BINs. Molecular and morphological results were congruent.

*Brachymeria *is distributed worldwide and includes about 307 described species (Noyes 2018). There are 46 described species in the Neotropical region (Noyes 2018) and at least 60 species awaiting description (unpublished data, MTT). Therefore, the *Brachymeria* fauna, sampled in Panguana, represents almost 10% of the Neotropical fauna.

### *Ceyxia *Girault, 1911

**Figure 7.**
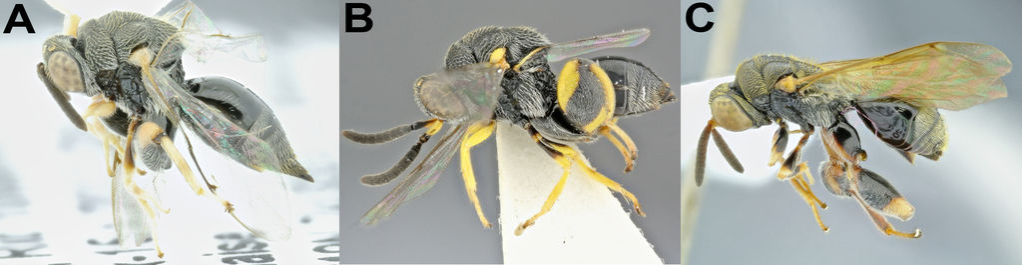
Diversity of the *Ceyxia *described species, females: **A.** *Ce. acutigaster*; **B.** *Ce. amazonica*; **C.** *Ce. bellissima*.

*Ceyxia* was represented by five species and six specimens collected only with the Malaise trap (Table 6). Six species had previously been recorded for Peru: *Ce. acutigaster*, *Ce. atuberculata*, *Ce. concitator*, *Ce. decreta*, *Ce. flaviscapus* and *Ce. villosa* (Andrade and Tavares 2009). We recovered *Ce. acutigaster* and *Ce. villosa* and another three species: *Ce. amazonica*, *Ce. bellissima* and *Ce. *sp1. Thus, eight species are now known for the genus in Peru. Specimens of *Ce. acutigaster*, *Ce. amazonica* and *Ce. bellissima* were submitted to the molecular pipeline, but no sequences were obtained (Fig. 7).

### *Stypiura *Kirby, 1883

**Figure 8.**
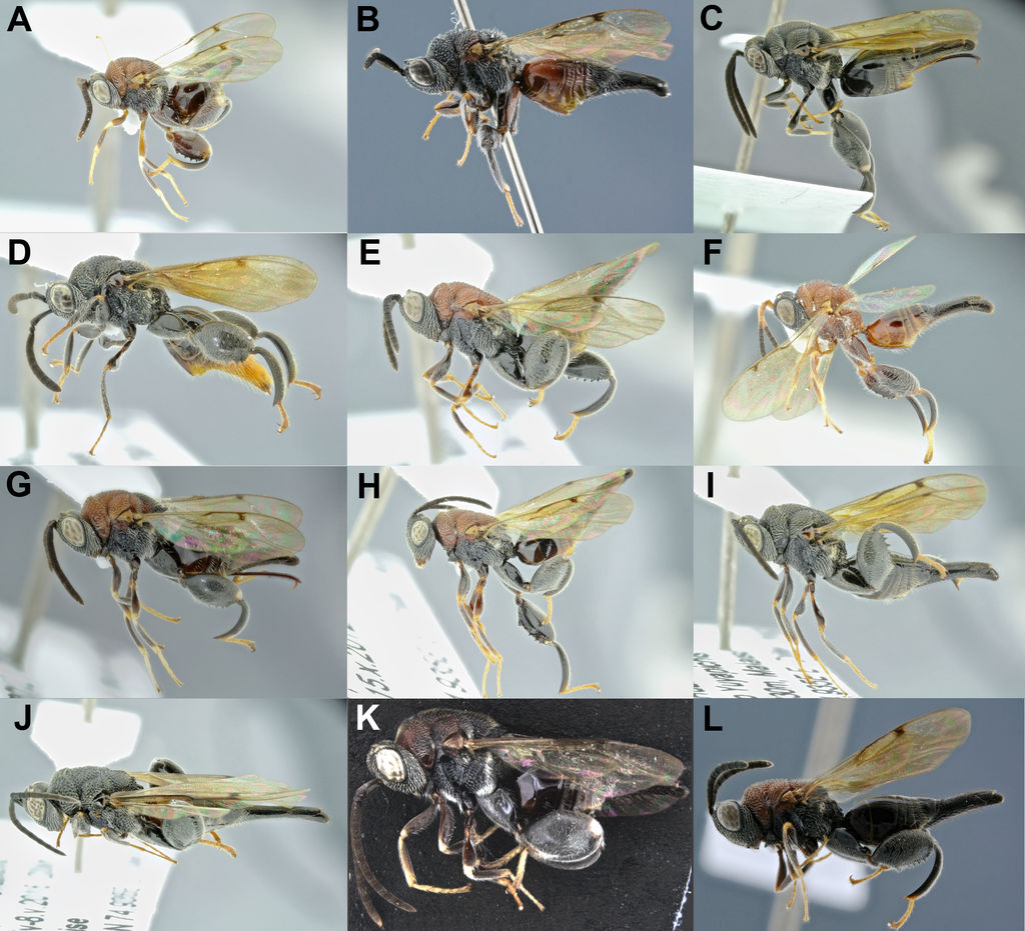
Diversity of *Stypiura*, females. **A.** *S. batesii*; **B.** *S.*sp1; **C.** *S. *sp2; **D.** *S.*sp3; **E.** *S.*sp4; **F. *S.*****sp5; **G.** *S.*sp6 (BOLD:ADF0812); **H.** *S.*sp7; **I.** *S.*sp8; **J.***S.*sp9; **K.** *S.*sp10 ; **L.** *S.*sp11.

A total of 18 specimens of *Stypiura* were obtained, representing 12 species (Table 7). According to Bouček (1992), amongst the genus of Phasgonophorini, only *Stypiura* had already been recorded from Peru. However, Bouček did not provide information on which species were found in Peru. In the present work, almost all recorded species are new. The only species found that had been previously described was *S. batesii*, which, so far, had not been recorded from Peru. Thus, 12 species are hereby recorded for that country: *S. batesii*, *S.* sp1, *S.* sp2, *S.* sp3, *S.* sp4, *S.* sp5, *S.* sp6, *S.* sp7, *S.* sp8, *S.* sp9, *S.* sp10 and *S.* sp11 (Fig. 8).

The most abundant species were *S.* sp6 and *S.* sp8, with 3 and 4 specimens, respectively. The species *S. batesii*, *S.* sp2, *S.* sp6 and *S.* sp7 were chosen for the molecular pipeline. Despite the cluster being congruent with the morphological delimitation of the species, the only species that received a BIN was *S.* sp6.

### *Dirhinus *Dalman, 1818

**Figure 9.**
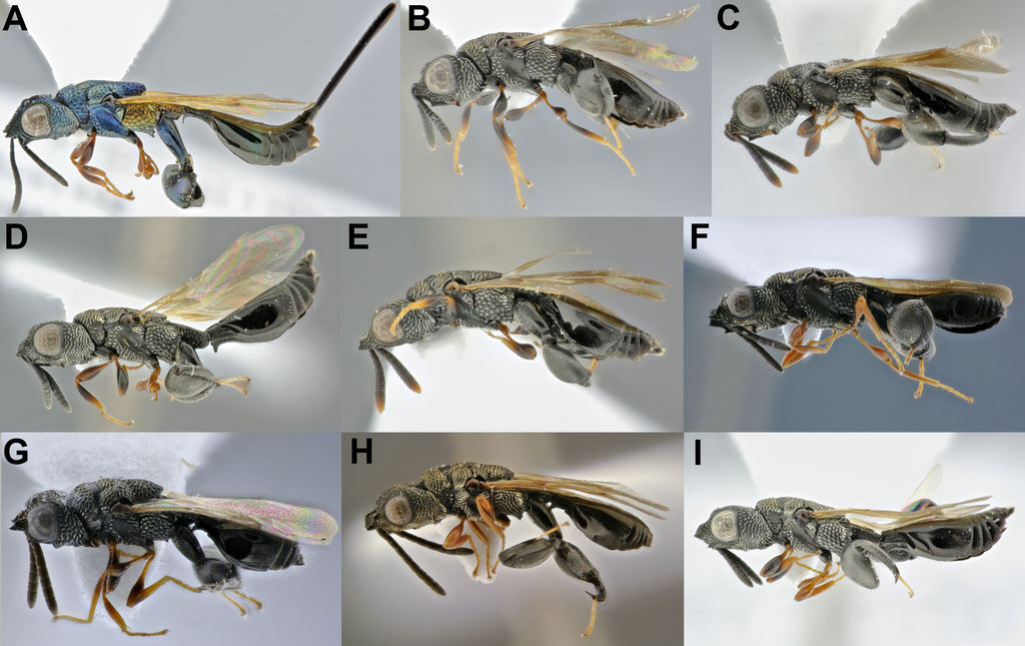
Diversity of *Dirhinus*, females, except F and H, males. **A.** *D. cameroni*; **B.** *D. buscki* (BOLD: ADF1234); **C.** *D. kirbyi*; **D.** *D.* sp1; **E.** *D.* sp2; **F.** *D. *sp3; **G.** *D.*sp4; **H.** *D.* sp5; **I.** *D.*sp6 (BOLD: ADE9797)

The current classification of *Dirhinus* presents three subgenera: *Dirhinus*, *Hontalia* and *Pareniaca*, all found in the New World; the second is exclusively neotropical. Although it is quite diverse for the New World (16 species), the only species known from Peru is *D. *(*Dirhinus*) *giffardii*, which was not found in the studied samples. After morphological analysis of the 28 specimens obtained, we were able to recognise nine species (Fig. 9). Eight species belong to subgenus *Pareniaca *(six new to science) and one species belongs to the subgenus Hontalia. We submitted 18 specimens, representing all species found of *Dirhinus,* to the molecular pipeline. Ten produced sequences longer than 100 bp and were included in a cluster analysis (Table 8). The clusters presented an average of 6.05% of nearest neighbour distance (NN), with the clustering fully corresponding with the results of the morphological analysis. Seven BINs were associated with the successfully sequenced samples, all new to BOLD (Table 8). These seven new species correspond to an increase of 43.7% in the *Dirhinus* diversity known from the New World.

### *Notaspidium *Dalla Torre, 1897

**Figure 10.**
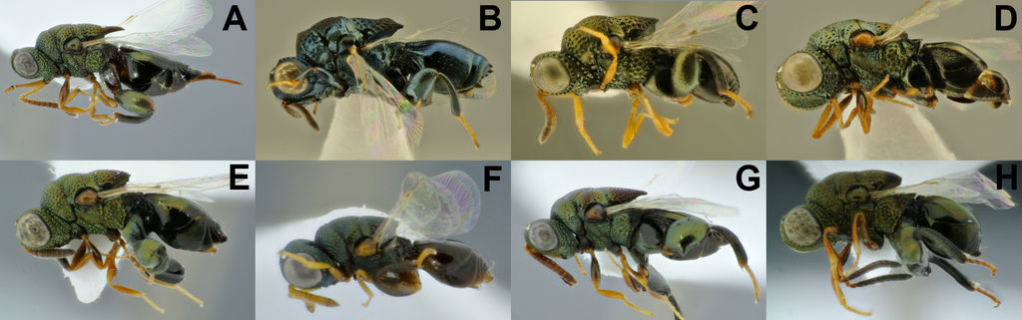
Diversity of the *Notaspidium *described species: **A.** *N. acutum*, female; **B.** *N. apantelis*, female*; ***C.** *N. boharti*, male;****D.** *N. braziliensis*; **E.** *N. burdicki*; **F.** *N. minutum*; **G.** *N. truncatum*; **H.** *N. villegsasi*

*Notaspidium *is the only genus of Haltichellinae previously recorded from Peru. It was known from two species (*N*. *apantelis* and *N. giganteum*). Only *N*. *apantelis*was obtained in the present study. *Notaspidium *was the dominant genus in the fogging samples, with 11 specimens and 8 species (Table 9). A total of 37 specimens and 12 species were found in the morphological analysis (Fig. 10), four of them new species. Eighteen specimens and 12 species were selected to the molecular pipeline. Eleven specimens produced sequences longer than 100 bp and were included in a cluster analysis (Table 2). The clusters presented an average of 11.26% nearest neighbour (NN) distance, fully matching the results of the morphological analysis. The four BINs found are new to BOLD (Table 9).

### *Aspirrhina *Kirby, 1883

**Figure 11.**
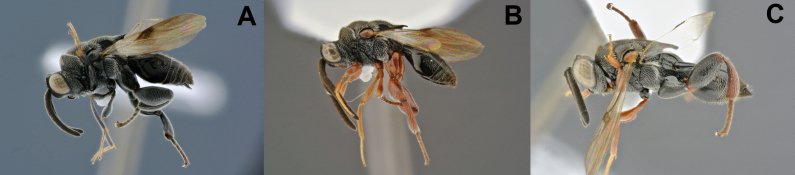
Diversity of *Aspirrhina*. **A.** *A. remotor*, male; **B.** *A. bifurca*, male; **C.** *A. dubitator*, female.

This is the first time that the genus *Aspirrhina *has been recorded from Peru. Three species (*A. remotor*,*A. bifurca* and *A. dubitator*) were identified, based on 12 specimens (Fig. 11). *Aspirrhina bifurca* was the most common species, with 8 specimens. Eight specimens representing all putative taxa found were submitted to the molecular pipeline, but no sequences were obtained.

### *Haltichella *Spinola, 1811

**Figure 12.**
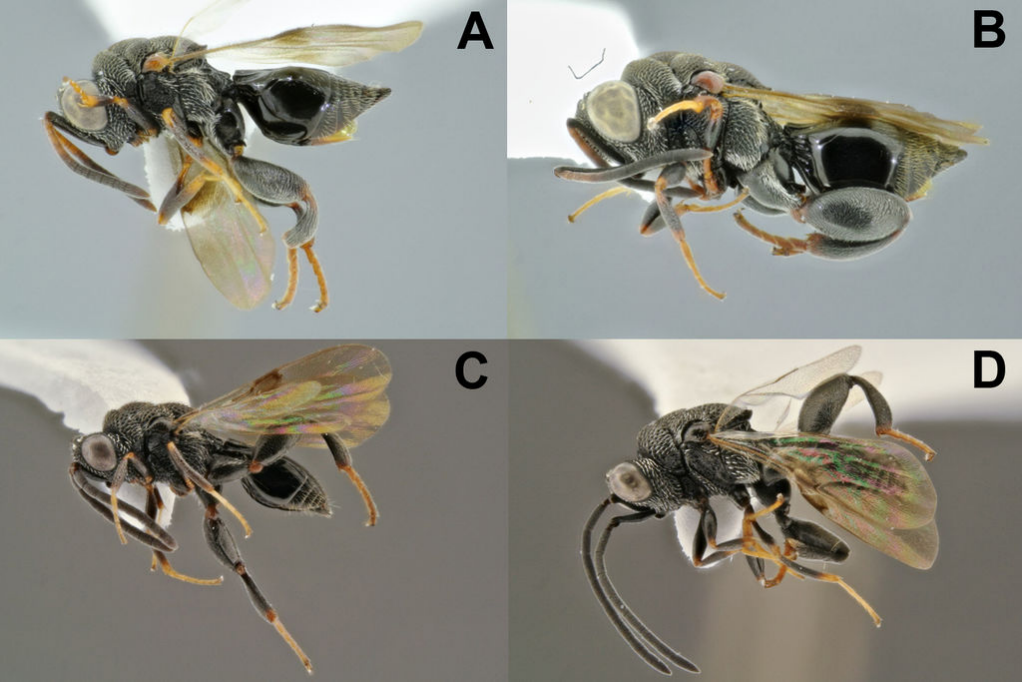
Diversity of *Haltichella*. **A.** *Ha. *sp1, female; **B.** *Ha. *sp2, female; **C.** *Ha. *sp3, male; **D.** *Ha.* sp4, male.

Although it is one of the most common genera obtained in neotropical Malaise trap samples, along with *Conura *and *Brachymeria*, this is the first time*Haltichella*has been recorded from Peru. Twenty-seven specimens were collected corresponding to four new species (Fig. 12). One species (*Ha. *sp3) was represented by 24 specimens and all others were singletons. Fourteen specimens were submitted to the molecular pipeline (Table 10). Only two specimens (representing *Ha. *sp3 and *Ha.* sp4) had the COI-5P fragment successfully amplified, but the sequences generated did not meet the criteria for BIN assignment. These specimens formed two separate clusters, which were congruent with the morphological analysis.

### *Hockeria *Walker, 1834

**Figure 13.**
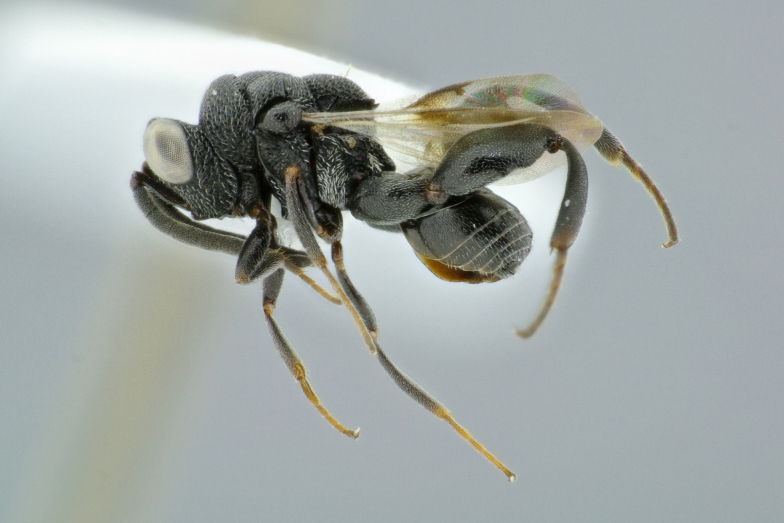
*Hockeria *sp1, male.

*Hockeria *is here recorded for the first time from Peru and was represented by two specimens of the same species (Fig. 13). Those two males do not seem to correspond to any known neotropical species. Both specimens were submitted to the molecular pipeline, but no sequences were obtained.

## Discussion

This paper presents the largest inventory ever made for the Peruvian Amazon fauna of Chalcididae and it provides new data which may drive future studies.

The abundance and diversity of species of Chalcididae in Panguana present similarities and divergences when compared with long term Malaise samplings from other Amazonian regions (housed at traditional Amazonian Institution in Brazil; unpublished data, MTT). *Conura* is usually the richest and more abundant genus in these samples, followed by *Brachymeria*, which is the case of the present samples. However, in the Panguana samples, *Melanosmicra*, *Notaspidium *and *Stypiura *were also diverse in species, similar to *Brachymeria*. *Melanosmicra *and*Stypiura* are usually captured in Malaise samples from the Amazonian forest, but they are not as diverse as in the Panguana samples. In forested areas, *Notaspidium* is not common in Malaise samples collected at soil level, although they are frequent in canopy samples obtained with interception traps (Malaise and window traps) and fogging. In Panguana, *Notaspidium* was well represented in the canopy samples, as well as at soil level. *Haltichella *tends to be frequent and abundant in samples of open vegetation (such as savannah and partially deforested areas), but not in forested areas. In Panguana, the genus was unexpectedly abundant, besides the relative high richness. All the above aspects indicate that the Chalcididae fauna of Panguana is peculiar and efforts should be made to better understand its fauna.

There are 386 Hymenoptera records publicly available on BOLD for Peru but none for Chalcididae. The 207 records presented here are the first records for Peruvian Chalcididae in the BOLD database. A total of 60 sequences were associated with a BIN, corresponding to 50 unique BINs, meaning that 81 specimens with sequences did not receive a BIN. This large number of specimens with sequences without a BIN was due to short sequences (around 401 bp). Given that most of those species are new to BOLD (and to science), in this case, the sequences were required to have a minimum of 500 bp to generate a new BIN in the system.

The sorted species, based on morphological data (morphospecies), were very consistent with those based on molecular data (DNA barcodes). While morphology indicated a total of 118 species, the barcodes indicated 121 species (sequences longer than 100 bp), which suggested three couples of cryptic species. This fact indicates that very few cryptic species are present in the sample and that both methods seem to be appropriate to study the diversity of Chalcididae fauna.

The 10 genera and 183 species obtained from the estimated 500 trap-days sampling effort demonstrate the high richness present in Panguana. Besides these species, one genus and twelve described species have been recorded to Peruvian Amazon (Table 1). Therefore, at least 11 genera and 195 species are present in Peruvian Amazon. This species richness is higher than the 149 species (described and undescribed) known from the State of Espírito Santo, Brazil (Tavares and de Araujo 2007), which is located in the Atlantic Forest, another biodiversity hotspot.

So far, only 7 genera and 11 described species had been recorded from the Peruvian Amazon. The present study adds 4 genera and 39 species, totalling 11 genera and 50 described species. Country-wise, the fauna of described species of Peruvian Chalcididae is now 13 genera and 76 described species, which is still less than 96 species reported from Colombia by Arias and Delvare (2003).

From the total of 183 species found here, 113 are potentially new species. Most of those species were represented by singletons, giving a clear picture of the potential diversity of the Peruvian Amazon and highlighting the importance of initiatives like the Panguana reserve.

We also draw attention to the importance of international collaboration initiatives, like the partnership established between ZSM and UFES, supporting the high-speed exchange of expertise and infrastructure, culminating in fast and high-quality results in taxonomy.

## Supplementary Material

3387F134-C99A-5A8B-892B-426B95E7CA5B 10.3897/BDJ.7.e35907.suppl1 Supplementary material 1 Peruvian Chalcididae summary table Data type: Excel sheet
File: oo_293928.xlsx https://binary.pensoft.net/file/293928 Bruno Cancian et al.

## Figures and Tables

**Table 1.** Checklist of formally described species of Chalcididae known to occur in Peru. Taxa marked with (*) are new records for the country. A question mark (?) indicates that no region or place in the country was recorded.

**Subfamily**	**Tribe**	**scientificName**	**Location**	**references**
Chalcidinae	-	-	-	-
-	Brachymeriini	-	-	-
-	-	*Brachymeria caudigera* Bouček, 1992*	Peruvian Amazon	present study
-	-	*B. costalimai* Delvare, 2017	Peruvian Amazon	Delvare et al. 2017, pg. 36
-	-	*B. flavipes* (Fabricius, 1793)	?	Arias and Delvare 2003, pg. 129
-	-	*B. mnestor* (Walker, 1841)*	Peruvian Amazon	present study
-	-	*B. mochica* Delvare, 2017	Pacific Coast	Delvare and Huchet 2017, pg. 46
-	-	*B. pandora* (Crawford, 1914)*	Peruvian Amazon	present study
-	-	*B. podagrica* (Fabricius, 1787)	Peruvian Amazon	Delvare and Huchet 2017, pg. 52
-	-	*Ceyxiaacutigaster*Andrade and Tavares, 2009	Peruvian Amazon	Andrade and Tavares 2009, pg. 515
-	-	*Ce. amazonica* Andrade and Tavares, 2009*	Peruvian Amazon	present study
-	-	*Ce. atuberculata* Andrade and Tavares, 2009	Peruvian Amazon	Andrade and Tavares 2009, pg. 518
-	-	*Ce. bellissima* Andrade and Tavares, 2009*	Peruvian Amazon	present study
-	-	*Ce. concitator*(Walker, 1862)	Peruvian Amazon	Andrade and Tavares 2009, pg. 522
-	-	*Ce. decreta* (Walker, 1862)	Peruvian Amazon	Andrade and Tavares 2009, pg. 523
-	-	*Ce. flaviscapus* Girault, 1911	Peruvian Amazon	Andrade and Tavares 2009, pg. 528
-	-	*Ce. villosa* (Olivier, 1791)	Peruvian Amazon	De Santis 1979, pg. 59
-	Chalcidini	-	-	-
-	-	*Conura abdominalis*(Walker, 1862)	?	Arias and Delvare 2003, pg. 133
-	-	*Co. acuta* (Fabricius, 1804)	?	Bouček 1992, pg. 13
-	-	*Co. adela* (Burks, 1939)*	Peruvian Amazon	present study
-	-	*Co. amoena* (Say, 1836)	Peruvian Amazon*	Arias and Delvare 2003, pg. 133
-	-	*Co. attacta*(Walker, 1864)	Peruvian Amazon*	Delvare 1992, pg. 133
-	-	*Co. bidentata* (Ashmead, 1904)*	Peruvian Amazon	present study
-	-	*Co. camescens* Delvare, 1992*	Peruvian Amazon	present study
-	-	*Co. chapadae* (Ashmead, 1904)*	Peruvian Amazon	present study
-	-	*Co. dares* (Walker, 1842)*	Peruvian Amazon	present study
-	-	*Co. debilis* (Say, 1836)*	Peruvian Amazon	present study
-	-	*Co. decisa* (Walker, 1861)	Peruvian Amazon*	Arias and Delvare 2003, pg. 134
-	-	*Co. destinata*(Walker, 1864)	Peruvian Amazon*	Arias and Delvare 2003, pg. 134
-	-	*Co. dorsimaculata* (Cameron, 1884)*	Peruvian Amazon	present study
-	-	*Co. eubule* (Cresson, 1865)	?	Delvare 1992, pg. 279
-	-	*Co. emarginata* (Fabricius, 1804)	?	Arias and Delvare 2003, pg. 134
-	-	*Co. expleta*(Walker, 1864)	?	Arias and Delvare 2003, pg. 134
-	-	*Co. femorata*(Fabricius, 1775)	Peruvian Amazon*	Arias and Delvare 2003, pg. 134
-	-	*Co. ferruginea*(Fabricius, 1804)	Peruvian Amazon*	Arias and Delvare 2003, pg. 134
-	-	*Co. flava* (Fabricius, 1804)	?	Arias and Delvare 2003, pg. 134
-	-	*Co. hirtifemora*(Ashmead, 1885)	?	Arias and Delvare 2003, pg. 131
-	-	*Co. immaculata* (Cresson, 1865)	Peruvian Amazon*	Delvare 1997, pg. 131
-	-	*Co. initia* Delvare, 1997	Peruvian Amazon	Delvare 1997, pg. 502
-	-	*Co. juxta* (Cresson, 1872)	?	Delvare 1997, pg. 135
-	-	*Co. laddi*(Girault, 1913)	?	Arias and Delvare 2003, pg. 135
-	-	*Co. maculipennis* (Cameron, 1884)	?	De Santis 1979, pg. 56
-	-	*Co. masus* (Walker, 1841)	?	Delvare 1992, pg. 284
-	-	*Co. miniata* (Cameron, 1884)*	Peruvian Amazon	present study
-	-	*Co. mourei*(De Santis, 1980)*	Peruvian Amazon	present study
-	-	*Co. nigrifrons* (Cameron, 1884)*	Peruvian Amazon	present study
-	-	*Co. pygmaea* (Fabricius, 1804)*	Peruvian Amazon	present study
-	-	*Co. rasplusi* Delvare, 1992*	Peruvian Amazon	present study
-	-	*Co. santaremensis* (Ashmead, 1904)*	Peruvian Amazon	present study
-	-	*Co. tricolorata* (Cameron, 1913)	?	Delvare 1992, pg. 318
-	-	*Co. vau* (Ashmead, 1904)*	Peruvian Amazon	present study
-	-	*Co. vesicula* Delvare, 1992*	Peruvian Amazon	present study
-	-	*Melanosmicra carenata* Navarro-Tavares and Tavares, 2008*	Peruvian Amazon	present study
-	-	*Me. flavicollis* (Cameron, 1904)*	Peruvian Amazon	present study
-	-	*Me. immaculata* Ashmead, 1904*	Peruvian Amazon	present study
-	-	*Me. nigra* Navarro-Tavares and Tavares, 2008*	Peruvian Amazon	present study
-	-	*Me. tricolor* Navarro-Tavares and Tavares, 2008*	Peruvian Amazon	present study
-	-	*Stenosmicra *sp.	?	Bouček 1992, pg. 98
-	Phasgonophorini	-	-	-
-	-	*Stypiurabatesii*(Kirby, 1883)*	Peruvian Amazon	present study
-	-	*S. dentipes* (Fabricius, 1804)	?	Arias and Delvare 2003, pg. 137
-	-	*S. serripes* (Fabricius, 1804)	?	Arias and Delvare 2003, pg. 138
Dirhininae	-	-	-	-
-	Dirhinini	-	-	-
-	-	*Dirhinus buscki* (Crawford, 1913)*	Peruvian Amazon	present study
-	-	*D. cameroni* (Ashmead, 1904)*	Peruvian Amazon	present study
-	-	*D. giffardii* Silvestri, 1913	?	De Santis 1979, pg. 68
-	-	*D. kirbyi* (Ashmead, 1904)*	Peruvian Amazon	present study
Haltichellinae	-	-	-	-
-	Haltichellini	-	-	-
-	-	*Aspirrhina bifurca* Halstead, 1991*	Peruvian Amazon	present study
-	-	*A. dubitator* (Walker, 1862)*	Peruvian Amazon	present study
-	-	*A. remotor*(Walker, 1862)*	Peruvian Amazon	present study
-	-	*Ecuadaproducta*Bouček, 1992	?	Bouček 1992, pg. 68
-	-	*Haltichella* spp.*	Peruvian Amazon	present study
-	-	*Hockeria *sp.*	Peruvian Amazon	present study
-	Hybothoracini	-	-	-
-	-	*Notaspidium acutum* Halstead, 1991*	Peruvian Amazon	present study
-	-	*N. apantelis*Bouček, 1992	Pacific Coast	Bouček 1992, pg. 73
-	-	*N. boharti*Halstead, 1991*	Peruvian Amazon	present study
-	-	*N. braziliensis*Halstead, 1991*	Peruvian Amazon	present study
-	-	*N. burdicki* Halstead, 1991*	Peruvian Amazon	present study
-	-	*N. minutum*Halstead, 1991*	Peruvian Amazon	present study
-	-	*N. truncatum*Halstead, 1991*	Peruvian Amazon	present study
-		*N. villegasi* Halstead, 1991*	Peruvian Amazon	present study
-	-	*N. giganteum*Halstead, 1991 *	Peruvian Amazon	Halstead 1991, pg. 233
-	-	*Zavoya brevispina* Bouček, 1992	Peruvian Amazon	Bouček 1992, pg. 59

**Table 2.** Cluster analysis of sequences obtained from 138 specimens, with minimum length of 100 bp (BIN: Barcode Index Number; NN Dist: distance to the nearest neighbour).

**OTU**	**materialSampleID**	**Genus**	**Species**	**BIN**	**NN Dist**
OTU-1	BC-ZSM-HYM-30556-H10	* Brachymeria *	sp11	N/A	16.031
OTU-2	BC-ZSM-HYM-27511-F04	* Brachymeria *	sp5	ADF1042	10.417
OTU-3	BC-ZSM-HYM-27511-F05	* Brachymeria *	sp10	ADF1766	10.417
OTU-3	BC-ZSM-HYM-30556-H06	* Brachymeria *	sp10	ADF1766	10.417
OTU-3	BC-ZSM-HYM-30556-H07	* Brachymeria *	sp10	ADF1766	10.417
OTU-4	BC-ZSM-HYM-25927-G12	* Brachymeria *	sp4	ADH9819	14.626
OTU-5	BC-ZSM-HYM-25927-G11	* Brachymeria *	sp7	N/A	10.549
OTU-6	BC-ZSM-HYM-25927-G05	* Brachymeria *	sp3	ADI0920	10.549
OTU-6	BC-ZSM-HYM-30556-H08	* Brachymeria *	sp3	ADI0920	10.549
OUT-7	BC-ZSM-HYM-30556-B05	* Brachymeria *	sp6	N/A	9.343
OUT-8	BC-ZSM-HYM-30556-B08	* Brachymeria *	* pandora *	N/A	9.343
OUT-8	BC-ZSM-HYM-30556-B09	* Brachymeria *	* pandora *	N/A	9.343
OUT-9	BC-ZSM-HYM-30556-B04	* Ceyxia *	sp1	N/A	12.121
OTU-10	BC-ZSM-HYM-30556-F02	* Conura *	sp62	N/A	9.848
OTU-11	BC-ZSM-HYM-30556-F05	* Conura *	sp65	N/A	7.323
OTU-12	BC-ZSM-HYM-30556-E11	* Conura *	sp59	N/A	6.818
OTU-13	BC-ZSM-HYM-30556-E12	* Conura *	sp60	N/A	9.848
OTU-14	BC-ZSM-HYM-30556-G05	* Conura *	sp78	ADO4114	78.283
OTU-15	BC-ZSM-HYM-30556-G06	* Conura *	sp79	N/A	10.354
OTU-16	BC-ZSM-HYM-30556-G03	* Conura *	sp75	N/A	7.323
OTU-17	BC-ZSM-HYM-30556-G04	* Conura *	sp77	N/A	10.744
OTU-18	BC-ZSM-HYM-30556-G01	* Conura *	sp73	N/A	6.061
OTU-19	BC-ZSM-HYM-30556-G02	* Conura *	sp74	N/A	8.081
OTU-20	BC-ZSM-HYM-30556-F11	* Conura *	sp71	N/A	7.576
OTU-21	BC-ZSM-HYM-30556-F12	* Conura *	sp72	N/A	9.091
OTU-22	BC-ZSM-HYM-30556-H05	* Conura *	* femorata *	N/A	10.499
OTU-23	BC-ZSM-HYM-30556-H03	* Conura *	*ferruginea*	N/A	8.192
OTU-24	BC-ZSM-HYM-30556-H01	* Conura *	sp84	N/A	8.267
OTU-25	BC-ZSM-HYM-30556-G10	* Conura *	sp81	N/A	11.616
OTU-26	BC-ZSM-HYM-30556-G09	* Conura *	* chapadae *	N/A	8.267
OTU-27	BC-ZSM-HYM-30556-G07	* Conura *	sp80	N/A	5.823
OTU-28	BC-ZSM-HYM-27511-F11	* Conura *	sp15	ADE9828	8.208
OTU-29	BC-ZSM-HYM-27511-G02	* Conura *	sp42	N/A	9.283
OTU-30	BC-ZSM-HYM-27511-F12	* Conura *	sp12	ADE9959	5.800
OTU-31	BC-ZSM-HYM-27511-G05	* Conura *	sp13	ADE9831	74.074
OTU-32	BC-ZSM-HYM-27511-G04	* Conura *	sp16	ADE9956	79.545
OTU-33	BC-ZSM-HYM-27511-G07	* Conura *	sp03	ADE9827	23.173
OTU-34	BC-ZSM-HYM-27511-G06	* Conura *	sp02	ADE9823	9.717
OTU-34	BC-ZSM-HYM-25927-C05	* Conura *	sp02	ADE9823	9.717
OTU-34	BC-ZSM-HYM-25927-D04	* Conura *	sp02	ADE9823	9.717
OTU-35	BC-ZSM-HYM-27511-G09	* Conura *	sp17	ADE9958	9.319
OTU-36	BC-ZSM-HYM-27511-G08	* Conura *	sp03	ADE9681	23.173
OTU-36	BC-ZSM-HYM-27511-H11	* Conura *	sp03	ADE9681	23.173
OTU-37	BC-ZSM-HYM-27511-G11	* Conura *	sp19	ADE9960	9.259
OTU-38	BC-ZSM-HYM-27511-G10	* Conura *	sp18	ADE9682	9.667
OTU-39	BC-ZSM-HYM-27511-H02	* Conura *	* nigrifrons *	ADE9829	74.074
OTU-40	BC-ZSM-HYM-27511-G12	* Conura *	sp20	ADE9957	7.317
OTU-41	BC-ZSM-HYM-27511-H04	* Conura *	sp90	ADE9826	5.800
OTU-42	BC-ZSM-HYM-27511-H03	* Conura *	sp18	ADE9822	9.667
OTU-43	BC-ZSM-HYM-27511-H07	* Conura *	sp22	ADE9825	8.333
OTU-44	BC-ZSM-HYM-27511-H08	* Conura *	* debilis *	ADE9824	11.787
OTU-45	BC-ZSM-HYM-27511-H05	* Conura *	sp21	ADE9830	11.295
OTU-46	BC-ZSM-HYM-27511-H06	* Conura *	sp06	ADE9680	25.126
OTU-47	BC-ZSM-HYM-25927-A07	* Conura *	sp04	ADI0628	10.452
OTU-47	BC-ZSM-HYM-25927-B01	* Conura *	sp04	ADI0628	10.452
OTU-48	BC-ZSM-HYM-25927-A08	* Conura *	sp05	ADI0820	8.333
OTU-49	BC-ZSM-HYM-25927-A02	* Conura *	* dares *	N/A	6.790
OTU-50	BC-ZSM-HYM-25927-A04	* Conura *	sp23	N/A	12.393
OTU-51	BC-ZSM-HYM-25927-A11	* Conura *	sp07	ADI1177	11.111
OTU-52	BC-ZSM-HYM-25927-B02	* Conura *	sp01	ADI0629	9.848
OTU-53	BC-ZSM-HYM-25927-A09	* Conura *	* camescens *	ADI0819	8.267
OTU-54	BC-ZSM-HYM-25927-A10	* Conura *	sp06	ADI0818	25.126
OTU-55	BC-ZSM-HYM-25927-B11	* Conura *	sp30	N/A	10.840
OTU-56	BC-ZSM-HYM-25927-C01	* Conura *	sp09	ADI0821	2.322
OTU-57	BC-ZSM-HYM-25927-B06	* Conura *	* amoena *	ADI0816	12.169
OTU-58	BC-ZSM-HYM-25927-B10	* Conura *	sp08	ADI0627	8.202
OTU-59	BC-ZSM-HYM-25927-C06	* Conura *	* pygmaea *	ADI0817	8.757
OTU-60	BC-ZSM-HYM-25927-C04	* Conura *	sp10	ADI1239	6.933
OTU-61	BC-ZSM-HYM-25927-C03	* Conura *	sp89	ADH8745	2.322
OTU-62	BC-ZSM-HYM-25927-C02	* Conura *	* adela *	ADI1049	8.429
OTU-63	BC-ZSM-HYM-25927-C09	* Conura *	sp11	ADI0626	8.800
OTU-64	BC-ZSM-HYM-25927-C07	* Conura *	sp32	N/A	6.962
OTU-65	BC-ZSM-HYM-25927-H09	* Conura *	sp14	ADI0822	9.649
OTU-66	BC-ZSM-HYM-30556-D09	* Conura *	* mourei *	N/A	1.032
OTU-67	BC-ZSM-HYM-30556-D08	* Conura *	sp47	N/A	9.366
OTU-68	BC-ZSM-HYM-30556-D11	* Conura *	sp49	N/A	11.111
OTU-69	BC-ZSM-HYM-30556-D10	* Conura *	sp48	N/A	7.323
OTU-70	BC-ZSM-HYM-30556-D02	* Conura *	sp35	N/A	7.576
OTU-70	BC-ZSM-HYM-30556-H02	* Conura *	* immaculata *	N/A	7.576
OTU-71	BC-ZSM-HYM-30556-D07	* Conura *	sp46	N/A	6.061
OTU-72	BC-ZSM-HYM-30556-D05	* Conura *	* vesicula *	N/A	8.642
OTU-73	BC-ZSM-HYM-30556-E08	* Conura *	sp57	N/A	7.163
OTU-74	BC-ZSM-HYM-30556-E07	* Conura *	sp56	N/A	78.283
OTU-75	BC-ZSM-HYM-30556-E10	* Conura *	* bidentata *	N/A	8.800
OTU-76	BC-ZSM-HYM-30556-E09	* Conura *	sp58	N/A	6.313
OTU-77	BC-ZSM-HYM-30556-E03	* Conura *	* dorsimaculata *	N/A	6.790
OTU-78	BC-ZSM-HYM-30556-D12	* Conura *	sp50	N/A	6.061
OTU-79	BC-ZSM-HYM-30556-E06	* Conura *	sp55	N/A	9.091
OTU-80	BC-ZSM-HYM-30556-E04	* Conura *	sp53	N/A	5.823
OTU-81	BC-ZSM-HYM-30556-F09	* Conura *	sp69	N/A	8.000
OTU-82	BC-ZSM-HYM-30556-F10	* Conura *	sp70	N/A	6.061
OTU-83	BC-ZSM-HYM-30556-F07	* Conura *	sp67	N/A	7.323
OTU-84	BC-ZSM-HYM-30556-F08	* Conura *	sp68	N/A	9.409
OUT-85	BC-ZSM-HYM-30556-C03	* Conura *	sp91	ADO1707	1.009
OUT-86	BC-ZSM-HYM-27511-C03	* Dirhinus *	* buscki *	ADF1234	28.620
OUT-86	BC-ZSM-HYM-27511-C09	* Dirhinus *	* buscki *	ADF1234	28.620
OUT-86	BC-ZSM-HYM-27511-C11	* Dirhinus *	* buscki *	ADF1234	28.620
OUT-86	BC-ZSM-HYM-25927-E04	* Dirhinus *	* buscki *	ADF1234	28.620
OTU-87	BC-ZSM-HYM-25927-E03	* Dirhinus *	sp1	N/A	8.000
OTU-88	BC-ZSM-HYM-27511-C04	* Dirhinus *	*kirbyi*	ADE9658	8.065
OTU-88	BC-ZSM-HYM-27511-C10	* Dirhinus *	*kirbyi*	ADE9658	8.065
OTU-89	BC-ZSM-HYM-30556-B01	* Dirhinus *	* cameroni *	N/A	15.301
OTU-90	BC-ZSM-HYM-30556-B03	* Dirhinus *	sp4	N/A	8.065
OTU-91	BC-ZSM-HYM-27511-C06	* Dirhinus *	sp6	ADE9797	28.620
OTU-92	BC-ZSM-HYM-30556-A03	* Haltichella *	sp4	N/A	13.889
OTU-93	BC-ZSM-HYM-30556-A02	* Haltichella *	sp3	N/A	15.051
OTU-94	BC-ZSM-HYM-27511-B01	* Melanosmicra *	* immaculata *	ADF0815	53.333
OTU-94	BC-ZSM-HYM-27511-B06	* Melanosmicra *	* immaculata *	ADF0815	53.333
OTU-94	BC-ZSM-HYM-27511-C01	* Melanosmicra *	* immaculata *	ADF0815	53.333
OTU-94	BC-ZSM-HYM-30556-C06	* Melanosmicra *	* immaculata *	N/A	53.333
OTU-95	BC-ZSM-HYM-27511-B02	* Melanosmicra *	sp3	ADF1337	22.727
OTU-95	BC-ZSM-HYM-30556-C01	* Melanosmicra *	sp3	ADF1337	22.727
OTU-96	BC-ZSM-HYM-27511-B03	* Melanosmicra *	sp1	ADF1769	48.387
OTU-97	BC-ZSM-HYM-27511-B11	* Melanosmicra *	*flavicolis*	ADE9942	10.840
OTU-99	BC-ZSM-HYM-30556-C05	* Melanosmicra *	* carenata *	N/A	8.586
OTU-100	BC-ZSM-HYM-30556-C08	* Melanosmicra *	* rugosa *	N/A	11.295
OTU-100	BC-ZSM-HYM-30556-C09	* Melanosmicra *	* rugosa *	N/A	11.295
OTU-101	BC-ZSM-HYM-30556-B12	* Melanosmicra *	* nigra *	N/A	22.727
OTU-101	BC-ZSM-HYM-30556-C07	* Melanosmicra *	* nigra *	N/A	22.727
OTU-102	BC-ZSM-HYM-30556-C02	* Melanosmicra *	* tricolor *	N/A	48.387
OTU-102	BC-ZSM-HYM-30556-C04	* Melanosmicra *	* tricolor *	N/A	48.387
OTU-103	BC-ZSM-HYM-27511-E03	* Notaspidium *	* minutum *	ADE9953	13.115
OTU-104	BC-ZSM-HYM-25927-E08	* Notaspidium *	* truncatum *	N/A	11.852
OTU-105	BC-ZSM-HYM-25927-E09	* Notaspidium *	sp2	ADJ2148	1.016
OTU-106	BC-ZSM-HYM-30556-A06	* Notaspidium *	sp1	N/A	13.333
OTU-107	BC-ZSM-HYM-30556-A12	* Notaspidium *	sp4	N/A	10.298
OTU-108	BC-ZSM-HYM-30556-A07	* Notaspidium *	* apantelis *	N/A	1.221
OTU-109	BC-ZSM-HYM-30556-A08	* Notaspidium *	* braziliensis *	N/A	1.221
OTU-110	BC-ZSM-HYM-30556-A10	* Notaspidium *	* burdicki *	N/A	83.951
OTU-111	BC-ZSM-HYM-30556-A11	* Notaspidium *	sp5	N/A	83.951
OTU-112	BC-ZSM-HYM-27511-D10	* Notaspidium *	* acutum *	ADF1074	1.368
OTU-113	BC-ZSM-HYM-27511-D11	* Notaspidium *	* boharti *	ADF1075	10.298
OTU-114	BC-ZSM-HYM-27511-E07	* Stypiura *	sp2	N/A	58.952
OTU-115	BC-ZSM-HYM-27511-E12	* Stypiura *	sp7	N/A	8.955
OTU-116	BC-ZSM-HYM-27511-F02	* Stypiura *	sp6	ADF0812	58.952
OTU-117	BC-ZSM-HYM-27511-F03	* Stypiura *	* batesii *	N/A	69.565
OTU-118	BC-ZSM-HYM-30556-C12	* Stypiura *	sp8	N/A	8.036

**Table 3.** *Conura *specimens, corresponding BINs and BOLD data of the specimens submitted to the molecular pipeline producing sequences longer than 200 bp.

**scientificName**	**Specimens**	**BIN**	**materialSampleID**
*Co. adela*	1	ADI1049	BC-ZSM-HYM-25927-C02
*Co. amoena*	1	ADI0816	BC-ZSM-HYM-25927-B06
*Co. bidentata*	1	N/A	BC-ZSM-HYM-30556-E10
*Co. camenscens*	1	ADI0819	BC-ZSM-HYM-25927-A09
*Co. chapadae*	1	N/A	BC-ZSM-HYM-30556-G09
*Co. dares*	2	N/A	BC-ZSM-HYM-25927-A02
*Co. debilis*	1	ADE9824	BC-ZSM-HYM-27511-H08
*Co. dorsimaculata*	1	N/A	BC-ZSM-HYM-30556-E03
*Co. femorata*	1	N/A	BC-ZSM-HYM-30556-H05
* Co. * *ferruginea*	1	N/A	BC-ZSM-HYM-30556-H03
*Co. immaculata*	1	N/A	BC-ZSM-HYM-30556-H02
*Co. mourei*	1	N/A	BC-ZSM-HYM-30556-D09
*Co. nigrifrons*	1	ADE9829	BC-ZSM-HYM-27511-H02
*Co. pygmaea*	1	ADI0817	BC-ZSM-HYM-25927-C06
*Co. vesicula*	1	N/A	BC-ZSM-HYM-30556-D05
*Co. *sp01	1	ADI0629	BC-ZSM-HYM-25927-B02
*Co. *sp02	3	ADE9823	BC-ZSM-HYM-25927-C05; BC-ZSM-HYM-25927-D04; BC-ZSM-HYM-27511-G06
*Co. *sp03	6	ADE9827; ADE9681	BC-ZSM-HYM-27511-G07; BC-ZSM-HYM-27511-G08; BC-ZSM-HYM-27511-H11
*Co. *sp04	2	ADI0628	BC-ZSM-HYM-25927-A07; BC-ZSM-HYM-25927-B01
*Co. *sp05	1	ADI0820	BC-ZSM-HYM-25927-A08
*Co. *sp06	2	ADI0818; ADE9680	BC-ZSM-HYM-25927-A10; BC-ZSM-HYM-27511-H06
*Co. *sp07	1	ADI1177	BC-ZSM-HYM-25927-A11
*Co. *sp08	1	ADI0627	BC-ZSM-HYM-25927-B10
*Co. *sp09	1	ADI0821	BC-ZSM-HYM-25927-C01
*Co. *sp10	1	ADI1239	BC-ZSM-HYM-25927-C04
*Co. *sp11	1	ADI0626	BC-ZSM-HYM-25927-C09
*Co. *sp12	3	ADE9959	BC-ZSM-HYM-27511-F12
*Co. *sp13	3	ADE9831	BC-ZSM-HYM-27511-G05
*Co. *sp14	1	ADI0822	BC-ZSM-HYM-25927-H09
*Co. *sp15	1	ADE9828	BC-ZSM-HYM-27511-F11
*Co. *sp16	1	ADE9956	BC-ZSM-HYM-27511-G04
*Co. *sp17	1	ADE9958	BC-ZSM-HYM-27511-G09
*Co. *sp18	2	ADE9682; ADE9822	BC-ZSM-HYM-27511-G10; BC-ZSM-HYM-27511-H03
*Co. *sp19	1	ADE9960	BC-ZSM-HYM-27511-G11
*Co. *sp20	1	ADE9957	BC-ZSM-HYM-27511-G12
*Co. *sp21	1	ADE9830	BC-ZSM-HYM-27511-H05
*Co. *sp22	1	ADE9825	BC-ZSM-HYM-27511-H07
*Co. *sp23	1	N/A	BC-ZSM-HYM-25927-A04
*Co. *sp30	1	N/A	BC-ZSM-HYM-25927-B11
*Co. *sp32	1	N/A	BC-ZSM-HYM-25927-C07
*Co. *sp35	7	N/A	BC-ZSM-HYM-30556-D02
*C. *sp42	1	N/A	BC-ZSM-HYM-27511-G02
*Co. *sp46	1	N/A	BC-ZSM-HYM-30556-D07
*Co. *sp47	1	N/A	BC-ZSM-HYM-30556-D08
*Co. *sp48	3	N/A	BC-ZSM-HYM-30556-D10
*Co. *sp49	3	N/A	BC-ZSM-HYM-30556-D11
*Co. *sp50	1	N/A	BC-ZSM-HYM-30556-D12
*Co. *sp53	1	N/A	BC-ZSM-HYM-30556-E04
*Co. *sp55	1	N/A	BC-ZSM-HYM-30556-E06
*Co. *sp56	1	N/A	BC-ZSM-HYM-30556-E07
*Co. *sp57	1	N/A	BC-ZSM-HYM-30556-E08
*Co. *sp58	1	N/A	BC-ZSM-HYM-30556-E09
*Co. *sp59	1	N/A	BC-ZSM-HYM-30556-E11
*Co. *sp60	1	N/A	BC-ZSM-HYM-30556-E12
*Co. *sp62	1	N/A	BC-ZSM-HYM-30556-F02
*Co. *sp65	1	N/A	BC-ZSM-HYM-30556-F05
*Co. *sp67	1	N/A	BC-ZSM-HYM-30556-F07
*Co. *sp68	1	N/A	BC-ZSM-HYM-30556-F08
*Co. *sp69	1	N/A	BC-ZSM-HYM-30556-F09
*Co. *sp70	1	N/A	BC-ZSM-HYM-30556-F10
*Co. *sp71	1	N/A	BC-ZSM-HYM-30556-F11
*Co. *sp72	1	N/A	BC-ZSM-HYM-30556-F12
*Co. *sp73	1	N/A	BC-ZSM-HYM-30556-G01
*Co. *sp74	1	N/A	BC-ZSM-HYM-30556-G02
*Co. *sp75	1	N/A	BC-ZSM-HYM-30556-G03
*Co. *sp77	1	N/A	BC-ZSM-HYM-30556-G04
*Co. *sp78	1	N/A	BC-ZSM-HYM-30556-G05
*C. *sp79	1	N/A	BC-ZSM-HYM-30556-G06
*Co. *sp80	1	N/A	BC-ZSM-HYM-30556-G07
*Co. *sp81	1	N/A	BC-ZSM-HYM-30556-G10
*Co. *sp84	1	N/A	BC-ZSM-HYM-30556-H01
*Co. *sp89	2	ADH8745	BC-ZSM-HYM-25927-C03
*Co. *sp90	8	ADE9826	BC-ZSM-HYM-27511-H04

**Table 4.** *Melanosmicra *specimens, BOLD data and corresponding BINs of the specimens submitted to the molecular pipeline.

**scientificName**	**Specimens**	**BIN**	**materialSampleID**
*M. areta*	5	ADF1337	BC-ZSM-HYM-27511-B02; BC-ZSM-HYM-27511-B09
*M. carenata*	2	N/A	BC-ZSM-HYM-27511-B04; BC-ZSM-HYM-27511-B10
*M. flavicollis*	1	ADE9942	BC-ZSM-HYM-27511-B11
*M. gracilis*	6	N/A	BC-ZSM-HYM-30556-B12; BC-ZSM-HYM-30556-C01
*M. immaculata*	2	ADF0815	BC-ZSM-HYM-27511-B06; BC-ZSM-HYM-27511-C01; BC-ZSM-HYM-27511-B01
*M. nigra*	19	N/A	BC-ZSM-HYM-30556-C06; BC-ZSM-HYM-30556-C07
*M. rugosa*	18	N/A	BC-ZSM-HYM-27511-B08; BC-ZSM-HYM-27511-B07; BC-ZSM-HYM-30556-C08; BC-ZSM-HYM-30556-C09
*M. tricolor*	2	N/A	BC-ZSM-HYM-30556-C02
*M. *sp1	1	ADF1769	BC-ZSM-HYM-27511-B03
*M. *sp2	5	N/A	BC-ZSM-HYM-27511-B12; BC-ZSM-HYM-30556-C04
*M.* sp3	3	N/A	BC-ZSM-HYM-30556-C05

**Table 5.** *Brachymeria *specimens, corresponding BINs and BOLD data of the specimens submitted to the molecular pipeline. An asterisk (*) indicates species collected using the fogging method.

**scientificName**	**Specimens**	**BIN**	**materialSampleID**
*B. caudigera*	1	N/A	BC-ZSM-HYM-25927-F10
*B. mnestor*	13	N/A	BC-ZSM-HYM-25927-G06; BC-ZSM-HYM-25927-G10; BC-ZSM-HYM-27511-F07
*B. pandora*	37	N/A	BC-ZSM-HYM-25927-G03; BC-ZSM-HYM-25927-F09
*B. *sp1	1*	N/A	N/A
*B. *sp2	1	N/A	BC-ZSM-HYM-27511-F09
*B. *sp3	2	ADI0920	BC-ZSM-HYM-25927-G11; BC-ZSM-HYM-25927-G05
*B. *sp4	1	ADH9819	BC-ZSM-HYM-25927-G12
*B. *sp5	2	ADF1042	BC-ZSM-HYM-27511-F04
*B. *sp6	3	N/A	BC-ZSM-HYM-25927-F07; BC-ZSM-HYM-25927-G04
*B. *sp7	1	N/A	BC-ZSM-HYM-25927-G11
*B. *sp8	3	N/A	BC-ZSM-HYM-25927-G02; BC-ZSM-HYM-25927-G01
*B. *sp9	1	N/A	BC-ZSM-HYM-25927-F06
*B. *sp10	1	ADF1766	BC-ZSM-HYM-27511-F05
*B*. sp11	1	N/A	BC-ZSM-HYM-30556-H10

**Table 6.** *Ceyxia *specimens and BOLD data of the specimens submitted to the molecular pipeline.

**scientificName**	**Specimens**	**BIN**	**materialSampleID**
*Ce. acutigaster*	1	N/A	BC-ZSM-HYM-27511-F06
*Ce. amazonica*	1	N/A	BC-ZSM-HYM-27511-A12
*Ce. bellissima*	1	N/A	BC-ZSM-HYM-27511-F10
*Ce. * * villosa *	1	N/A	N/A
*Ce. *sp1	1	N/A	N/A

**Table 7.** *Stypiura *specimens, coprresponding BINs and BOLD data of the specimens submitted to the molecular pipeline.

**scientificName**	**Specimens**	**BIN**	**materialSampleID**
*S. batesii*	1	N/A	BC-ZSM-HYM-27511-F03
*S. *sp1	1	N/A	BC-ZSM-HYM-25927-E11
*S. *sp2	1	N/A	BC-ZSM-HYM-27511-E07
*S. *sp3	1	N/A	BC-ZSM-HYM-27511-E10
*S. *sp4	1	N/A	BC-ZSM-HYM-27511-E08
*S. *sp5	1	N/A	BC-ZSM-HYM-27511-E09
*S. *sp6	1	BOLD:ADF0812	BC-ZSM-HYM-27511-F02; BC-ZSM-HYM-25927-E12; BC-ZSM-HYM-30556-C11
*S. *sp7	1	N/A	BC-ZSM-HYM-27511-E12
*S. *sp8	1	N/A	BC-ZSM-HYM-27511-F01; BC-ZSM-HYM-25927-F02; BC-ZSM-HYM-25927-F03; BC-ZSM-HYM-30556-C12
*S. *sp9	1	N/A	BC-ZSM-HYM-27511-E11
*S. *sp10	1	N/A	N/A
*S. *sp11	3	N/A	BC-ZSM-HYM-25927-F01; BC-ZSM-HYM-30556-C10

**Table 8.** *Dirhinus *specimens, corresponding BINs and BOLD data of the specimens submitted to the molecular pipeline.

**scientificName**	**Specimens**	**BIN**	**materialSampleID**
* D. * * buscki *	4	ADF1234	BC-ZSM-HYM-27511-C03; BC-ZSM-HYM-27511-C09; BC-ZSM-HYM-27511-C11
* D. * * cameroni *	8	N/A	BC-ZSM-HYM-25927-E02; BC-ZSM-HYM-27511-C02; BC-ZSM-HYM-30556-B01
* D. * *kirbyi*	4	ADE9658	BC-ZSM-HYM-27511-C04; BC-ZSM-HYM-27511-C08; BC-ZSM-HYM-27511-C10
*D.* sp1	4	N/A	BC-ZSM-HYM-25927-E03; BC-ZSM-HYM-27511-C07; BC-ZSM-HYM-30556-B02
*D.* sp2	1	N/A	BC-ZSM-HYM-25927-E05
*D.* sp3	1	N/A	BC-ZSM-HYM-25927-E04
*D.* sp4	4	N/A	BC-ZSM-HYM-30556-B03
*D.* sp5	1	N/A	BC-ZSM-HYM-27511-C05
*D.* sp6	1	ADE9797	BC-ZSM-HYM-27511-C06

**Table 9.** *Notaspidium *specimens, corresponding BINs and BOLD data of the specimens submitted to the molecular pipeline. Brackets indicate the number of specimens collected using the fogging method.

**scientificName**	**Specimens**	**BIN**	**materialSampleID**
*N. acutum*	5	ADF1074	BC-ZSM-HYM-27511-D10
*N. apantelis*	1(1)	N/A	BC-ZSM-HYM-30556-A07
*N. boharti*	2(1)	ADF1075	BC-ZSM-HYM-30556-A11 BC-ZSM-HYM-27511-D11
*N. braziliensis*	2(2)	N/A	BC-ZSM-HYM-30556-A08
*N. burdicki*	13(2)	N/A	BC-ZSM-HYM-27511-E01 BC-ZSM-HYM-27511-D12 BC-ZSM-HYM-25927-E07 BC-ZSM-HYM-25927-E06
*N. minutum*	3(1)	ADE9953	BC-ZSM-HYM-27511-E03
*N. truncatum*	2	N/A	BC-ZSM-HYM-25927-E08 BC-ZSM-HYM-27511-E04
*N. villegasi*	1	N/A	BC-ZSM-HYM-27511-E05
*N.*sp1	3(2)	N/A	BC-ZSM-HYM-30556-A09
*N.*sp2	2	ADJ2148	BC-ZSM-HYM-27511-E06 BC-ZSM-HYM-25927-E09
*N. *sp3	1(1)	N/A	BC-ZSM-HYM-30556-A12
*N.*sp4	2(1)	N/A	BC-ZSM-HYM-25927-E10

**Table 10.** *Haltichella *specimens, corresponding BINs and BOLD data of the specimens submitted to the molecular pipeline.

**scientificName**	**Specimens**	**BIN**	**materialSampleID**
*H. *sp1	1	N/A	BC-ZSM-HYM-27511-D02
*H. *sp2	24	N/A	BC-ZSM-HYM-27511-D09; BC-ZSM-HYM-27511-D08; BC-ZSM-HYM-27511-D07; BC-ZSM-HYM-27511-D06; BC-ZSM-HYM-27511-D03; BC-ZSM-HYM-27511-D04; BC-ZSM-HYM-27511-D05; BC-ZSM-HYM-25927-D08; BC-ZSM-HYM-25527-D09; BC-ZSM-HYM-25927-D10; BC-ZSM-HYM-30556-A01
*H. *sp3	1	N/A	BC-ZSM-HYM-30556-A02
*H. *sp4	1	N/A	BC-ZSM-HYM-30556-A03
